# Prevalence of trochlear dysplasia in an 1162 retrospective cohort study using CT scans

**DOI:** 10.1186/s12891-024-07579-8

**Published:** 2024-07-17

**Authors:** Vella-Baldacchino Martinique, Cipolla Alessandra, Guy Sylvain, LiArno Sally, Faizan Ahmad, Argenson Jean-Noel, Ollivier Matthieu

**Affiliations:** 1 Department of Surgery & Cancer, MSk Lab – Imperial College London, Sir Michael Uren Hub, 86 Wood Ln, London, W12 0BZ UK; 2 AP-HM, Sainte-Marguerite Hospital, Institute for Locomotion, Department of Orthopedics and Traumatology, Aix-Marseille University, CNRS, ISM, Marseille, France; 3 Department of Orthopedics and Traumatology, Institute of Movement and Locomotion, St. Marguerite Hospital, 270, boulevard Sainte-Marguerite, BP 29, Marseille, 13274 France; 4 University of Turin, CTO Hospital (C.T.O. Centro Traumatologico Ortopedico), Via Gianfranco Zuretti, 29, Torino, 10126 TO Italy; 5 Stryker Orthopaedics, Mahwah, NJ USA

**Keywords:** Knee, Patella, Patellofemoral, Trochlear dysplasia

## Abstract

**Hypothesis/purpose:**

The prevalence of trochlear dysplasia is common in different populations.

**Background:**

The prevalence of trochlear dysplasia in the general population, categorised by sex, race, age, and body mass index, has been sparse. This study aimed to define the prevalence of trochlear dysplasia based on the latter categories.

**Study design:**

Cohort retrospective study.

**Methods:**

1162 skeletal mature healthy femora were obtained from a CT-scan-based modelling system (SOMA). Thin slice CT scans were acquired exclusively for medical indications such as polytrauma (20%), CT angiography (70%) and other reasons (i.e. Total Joint Replacement) (10%). Trochlear dysplasia was measured using Pfirmann’s method. Patient demographics such as age, race and sex were recorded.

**Results:**

The overall prevalence of trochlear dysplasia is 4.5% and is far more common in Asian female patients compared to Caucasian, African and Middle Eastern knees.

**Conclusion:**

Overall, the prevalence of dysplasia in the general population was determined to be 4.5%, with female patients being more likely to suffer from the condition. Patients of Asian and Caucasian race were more likely to have trochlear dysplasia, while Middle Eastern male patients displayed more dysplastic values than their female counterparts.

## Introduction

Femoral trochlear dysplasia is an abnormality in the shape and depth of the trochlea groove [1]. The trochlea groove keeps the patella reduced above 30° of flexion, acting **as** one of the primary stabilisers of the patellofemoral joint [2]. Below 30°, the main stabiliser is the medial patellofemoral ligament [2]. Trochlear dysplasia is a predisposing factor causing patellar instability [3]. Dysplasia increases the likelihood of patella dislocation laterally at the first 30’ of flexion as the patella fails to engage into the native groove [4, 5]. Fifty-five per cent of patients with patellofemoral osteoarthritis have trochlear dysplasia, with patients commonly complaining of anterior knee pain [1, 4, 6].

Trochlear dysplasia was first described in 1941 by Knuttson, followed by Maldague and Malghem, using strictly lateral conventional radiographs (CR) [7, 8]. Henri Dejour classified trochlear dysplasia into Types I to III based on the crossing sign observed on lateral radiographs [9]. David Dejour further established a four-step classification using axial CT scans [10]. The Dejour classification is thought to be the gold standard in the literature. The two-tier classification system, using only low-grade (type A) and high-grade trochlear dysplasia (types B–D), can be used to reliably classify dysplasia rather than the 4-grade classification which showed fair inter-observer and intra-observer reliability [2, 11–13]. A recent literature review has shown that since 1990, 46 unique measurements using X-ray, CT and MRI modalities have been published to describe trochlear dysplasia [4]. Different approaches to assessing dysplasia and a lack of international consensus on which measurement or classifications to use have resulted in the inability to apply transferable treatment algorithms between each institution [4].

The sulcus angle is a trochlea measurement that can quickly and reproducibly be measured [14]. It is defined by the intersection of the lines connecting the highest point of the femoral condyles to the deepest point of the trochlea groove [5]. The trochlea has physiologic concavity with a normal angle of 135 ± 10◦, and a cutoff value of 145 degrees was introduced [2, 15]. However, the sulcus angle has limitations as it is unable to describe side-to-side differences in the inclination of the medial and lateral trochlea facets [16]. Alternatively, Pfirmann introduced a separate MRI method to measure trochlea depth, which could differentiate trochlea dysplastic knees from normal knees with a sensitivity of 100% and a specificity of 96% [17]. The trochlear depth was assessed using the medial and lateral femoral condyles and the distance between the deepest point of the groove and the line parallel to the posterior outlines of the femoral condyles [17]. Pfirmann demonstrated that MRIs are more advantageous than radiographs because they can visualise cartilage at the joint surface in greater detail using the trochlear depth method [17].

The prevalence of trochlear dysplasia varies between 0.7 and 2% but is present in up to 85% of patients with patellar instability [1, 2, 18]. The prevalence of trochlear dysplasia in the general population, categorised by sex, race, age, and body mass index, has been sparse. This study aimed to define the prevalence of trochlear dysplasia based on the latter categories using CT scan measurements.

## Methods

1162 skeletal mature healthy femora were retrieved from a CT scan-based modelling and analysis system (SOMA, Stryker, Mahwah, New Jersey) [19]. All scans were obtained per local legal and regulatory requirements, including ethics board approval and informed consent from all subjects and/or their legal guardians. The SOMA database comprises over 25,000 bone models obtained from over 3,600 patients worldwide. Thin slice CT scans were acquired exclusively for medical indications such as polytrauma (20%), CT angiography (70%) and other reasons (i.e. Total Joint Replacement) (10%), pixel spacing: Median: 0.78 mm, IQR: 0.14 mm, Slice Spacing: Median: 1.00 mm, Interquartile Range: 0.20 mm) [19–22]. . Subjects with bone or joint abnormalities, substantial osteoarthritis or evidence of previous surgery were excluded before CT scan selection through radiographic inspection. All CT scans were segmented with standard software (MeVisLab and Materialise Mimics) according to a standardised protocol [19, 22]. SOMA automatically transfers measurements defined on an averaged 3D-bone template based on the available 1070 datasets to each dataset [19, 22]. This ensures highly accurate and reproducible measurements across a large population [19, 22]. Subjects were positioned with their knee extended, and all measurements were taken using a semi-automated measuring system 3 cm from the joint line. This study was conducted following approval of the research protocol by the local ethical committee (Aix-Marseille University) performed **i**n accordance with relevant guidelines and regulations, and the research was carried out in compliance with the Helsinki Declaration.

**Fig. 1:**
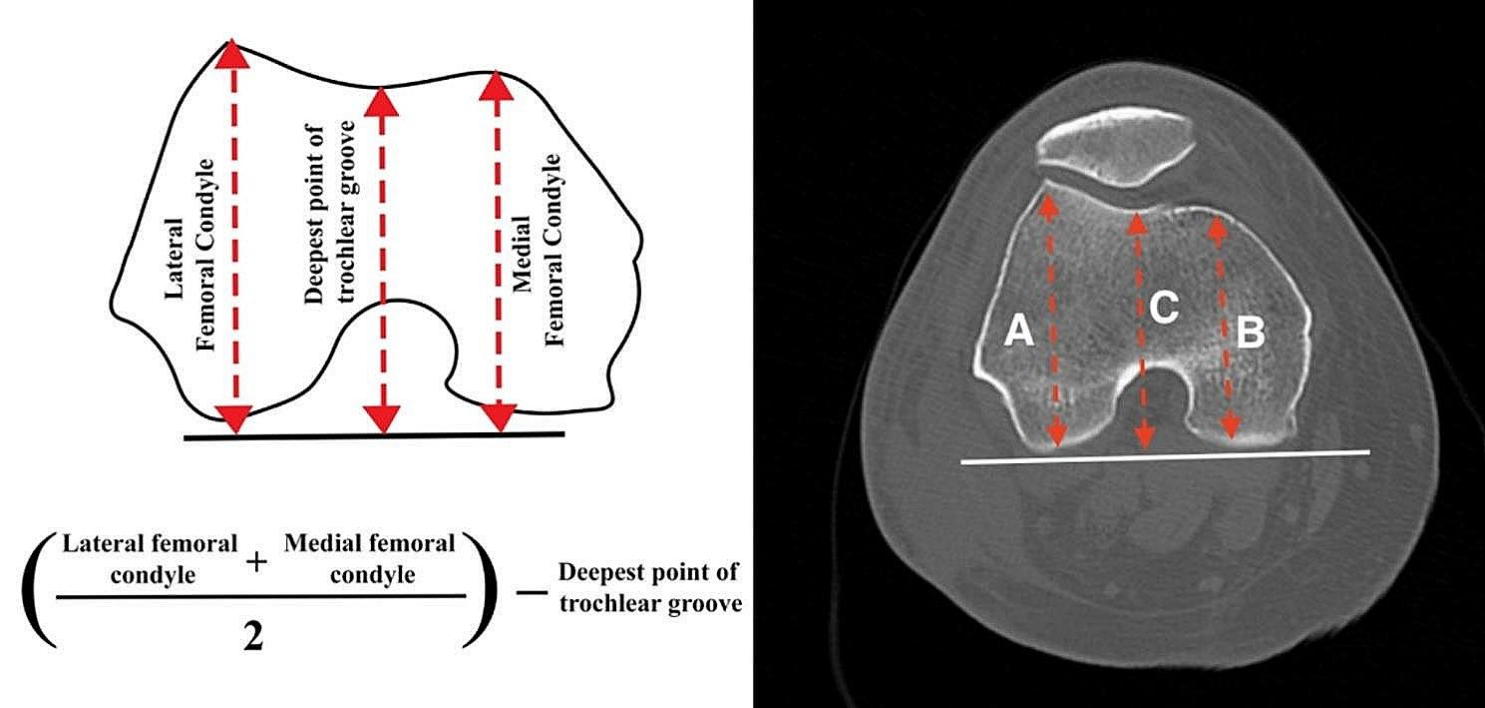
A schematic line illustration and images from the CT scan-based modelling and analysis system. Trochlear depth was measured using the largest anterior-posterior diameter of the lateral (**A**) and medial (**B**) femoral condyle and the deepest points of the trochlea groove (**C**) perpendicular to the posterior condyle. This is in accordance with Pfirmann method using the formula (A + B/2) – C

Height, weight, body mass index, sex, race and age are all in the institution’s database and linked to the CT scan images. The images were then analysed to measure the trochlea depth. Trochlea depth was measured using the largest anterior-posterior diameter of the medial and lateral femoral condyle. The deepest point of the trochlea groove was measured perpendicular to the posterior femoral condyle 3 cm above the joint line. A visual description of the method may be found in Fig. 1. This is in accordance with Pfirmann method [1].

Basic patient demographics were reported, as seen in Table 1. Qualitative variables were reported as numbers, and quantitative variables were mean ± standard deviation. Percentiles were used to classify a knee as dysplastic, where high grade dysplasia was defined as values at below the 5th centile, low grade dysplasia 5th – 25th centile and normal morphology as above the 5th centile. A statistical analysis using the Chi-square test and Fisher exact test, where appropriate, was conducted using STATA (version 18; StataCorp LLC, Texas, USA.) [23]. The significance threshold was set at 0.05.

**Table 1:** Cohort characteristics

Parameter	Summary Statistic
**Number of patients (knees)**	1162
**Female (number of patients)**	568
**Mean Age for each Race category (SD)**	
Caucasian	62 (15)
Asian	66 (19)
African	51 (20)
Middle Eastern	49 (13)
**Race categories (number of patients)**	
Caucasian	666
Asian	463
African	16
Middle Eastern	16
**Trochlea depth (mm)**	
Mean (SD)	5.2 (1.3)
Range	0.6–9.2
1st quartile – Lower	4.3
2nd quartile – Median	5.15
3rd quartile - Upper	6.1

## Results

As seen in Table 1, there were 1162 patients, and the mean age of the study population was 63 years. There were 568 female knees and 590 male knees, 4 knees had sex reported as missing. The study population consisted of 666 Caucasian knees, followed by Asian (*n* = 463), African (*n* = 16) and Middle Eastern patients (*n* = 16). Mean values are presented as they showed evidence of a normal distribution pattern for all ethnicities when using box plots which were symmetrical with the median line at the centre of the box [24]. The mean trochlear depth using Pfirmann’s method was 5.2 mm. High-grade dysplasia was classified as values at or below the 5th percentile and low-grade dysplasia within the 5th -25th percentile. 52 patients (4.5%) had dysplastic trochleae with a trochlea depth of less than 3 mm. Most of these patients were female (*n* = 38) compared to male patients (*n* = 29). The Chi-square test showed that females are associated with more dysplastic trochlea (*p* = 0.01). The Pearson correlation between dysplasia and sex was moderate and positive (*r* = 0.4, *p* = 0.01). This was true in Caucasian and Asian female patients (*p* = 0.01). However, no association was found in the African cohort (*p* = 0.40). Middle Eastern male patients demonstrated more dysplastic values than their female counterparts; however, there was no association (*p* = 0.15). Overall, the prevalence of trochlear dysplasia was higher among Asian and Caucasian ethnic groups (Table 2).

**Table 2:** Prevalence of trochlear dysplasia as defined by percentiles. Normal is defined as values above the 25th percentile. Low grade – within the 5th to 25th percentile. High grade: below the 5th percentile

Trochlea depth (mm)	Trochlear dysplasia					
	Normal Greater than 25th ^centile^		Low Grade Dysplasia 25th		High-Grade Dysplasia	
	Male	Female	Male	Female	Male	Female
Caucasian	5.8	4.9	5.0,	4.1	3.9	3.0
Number of patients.	261	223	80	65	16	21
African	**6.3**,	**5.2**	**5.4**	**4.6**	**4.2**	**3.4**
Number of patients	5	7	1	2	0	1
Asian	5.3,	4.4	4.5	3.7	3.3	2.6
Number of Patients	158	192	39	46	12	15
Middle Eastern	6.0,	4.8	5.0	4.4	3.6	4.2
Number of patients	10	1	3	0	1	1

## Discussion

The main finding of the study was, the overall prevalence of trochlear dysplasia was 55 out of 1162 knees (4.5%) with a value, according to the Pfirmann method, of less than 3 mm. Similar to other studies, females are more likely to be dysplastic except in Middle Eastern patients, where males had higher values; however, this was not significant *p* > 0.05. The female Asian population has a higher prevalence of high-grade dysplasia compared to other ethnicities.

The only two studies describing the prevalence of trochlear dysplasia were based on a smaller group of patients, 420 and 16 patients, respectively [27]. Healthy femora were only included in the study of Pfirmann et al., whilst the study of Greslamer et al. included all patients with knee pain [27]. Pfirmann included MRI-imaged femora that had already been confirmed as dysplastic, as this was a comparison study, whilst Greslamer used dry bones in a radiographic-based study [27].

For both studies, published before 2000, the primary outcome was determining the most reliable method to measure trochlear dysplasia, reporting the prevalence as a secondary outcome. Our study is the first to analyse over 1000 healthy knees, with the prevalence of trochlear dysplasia in different sexes and ethnicities as the primary outcome. This is the most extensive study ever published describing trochlear dysplasia and the first describing the pathology in different ethnicities. Two other studies have highlighted the need to analyse variations in various ethnic populations and sexes [25, 26].

Trochlear dysplasia is one of the essential mechanical factors causing patellofemoral pain, patellar instability and permanent damage to the patellofemoral cartilage, triggering patellofemoral osteoarthritis [27]. Patellofemoral dysplasia remains challenging to manage due to its controversial aetiology, complex biomechanical behaviour, and lack of universally accepted guidelines for the correct treatment [28]. Often, trochlear dysplasia requires surgical correction when symptomatic [16]. This study highlights the importance of trochlear dysplasia, representing the underlying cause in 96% of patients with recurrent patellar instability worldwide [29]. . Apart from dysplasia, patella dislocations are associated with injury to medial patellofemoral ligament, increased tibial tubercle-trochlear groove distance and patella alta [30, 31]. Dislocations that recur frequently damage the patellofemoral articular cartilage, causing patellofemoral osteoarthritis and ultimately disabling patients. Depending on the surgeon and patient’s preference, patellofemoral osteoarthritis may be treated surgically with a total knee or patellofemoral joint replacement [32]. With no gold standard treatment for patellofemoral cartilage degeneration, treatment remains challenging. Therefore, research on trochlear dysplasia remains essential. As a result of this study, future power calculations can be carried out for specific cohorts, and the race of patients who need to be recruited can be established. Future research should include sex and race variations to evaluate a broad spectrum of trochlea anatomy [33].

## Limitations

Measurements were taken from CT scans where the knee was placed in an extended position, limiting us to Pfirmanns method. The knee must be flexed to obtain a sulcus angle [18]. In this study, we were unable to determine if patients had a history of previous patella dislocations, which may explain a slightly higher prevalence of dysplastic trochlea in this study compared to Pfrimann et al.‘s study associated with 2% [1]. The number of patients in the Middle Eastern group was smaller than that of Caucasians and Asians. This underpowered sample may be the reason why male patients were more likely to have dysplastic conditions in the Middle Eastern group compared with Caucasian and African patients. Despite such a large cohort of over 1000 knees, Middle Easterns and Africans were still underrepresented, emphasising the need for more efforts to be made in the future to recruit subsets of these populations. As this was a retrospective cohort study, a sample size calculation was not performed.

## Conclusion

Research has focused on classification systems for trochlear dysplasia detection and treatment. Without knowing the true prevalence of the disease in the population, research is likely to remain focused on other major joint diseases. Knowing an overall prevalence of at least 4.5% in the general population shows that patellofemoral research should remain a priority for continuous research development.

## Data Availability

The datasets used and/or analysed during the current study available from the corresponding author on reasonable request.

## Declarations

### Level of evidence

Prognostic Level III.

## Abbreviations

BMI: Body mass index
TT-TG: Tibial tuberosity–trochlea groove
SOMA: Stryker Orthopaedics Modeling and Analytics ()
